# Maternal oral supplementation with *Saccharomyces boulardii* I-1079 during gestation and early lactation impacts the early growth rate and metabolic profile of newborn puppies

**DOI:** 10.3389/fnut.2025.1500600

**Published:** 2025-02-27

**Authors:** Ilyas Bendahmane, Quentin Garrigues, Emmanuelle Apper, Amélie Mugnier, Ljubica Svilar, Jean-charles Martin, Sylvie Chastant, Annabelle Meynadier, Hanna Mila

**Affiliations:** ^1^NeoCare, Université de Toulouse, ENVT, Toulouse, France; ^2^GenPhySE, Université de Toulouse, Institut national de recherche pour l'agriculture, l'alimentation et l'environnement (INRAE), École nationale vétérinaire de Toulouse (ENVT), Castanet Tolosan, France; ^3^Lallemand SAS, Blagnac, France; ^4^CriBioM, Aix Marseille Université, Marseille, France

**Keywords:** nutritional programming, early growth, proline, metabolome, newborn dog, dog, yeast

## Abstract

Nutritional programming is a manipulation of fetal and neonatal development through maternal feeding. In humans and pigs, maternal yeast supplementation was demonstrated as a promising approach to positively to modulate newborns' health. This study aimed to investigate the effects of *Saccharomyces cerevisiae* var. *boulardii* CNCM I-1079 (SB) supplementation in pregnant and lactating bitches on the newborns' early growth rate (EGR, between birth and 2 days of life), metabolic profiles, and the association between both of them. A total of 17 female dogs and their 81 puppies were included. From day 28 of gestation until the end of the study, bitches were divided into two groups, one of which received orally 1.3 × 10^9^ colony forming units of live yeast per day. Puppies from mothers receiving the live yeast were defined as the SB group (*n* = 40) and the others were defined as the placebo group (*n* = 4 1). For each puppy, EGR was calculated, and blood and urine samples were collected at D2 for metabolome analysis using liquid chromatography-mass spectrometry (LCMS). Puppies from the SB group presented higher EGR compared with the placebo group (12% vs. 7%; *p* = 0.049). According to the Sparse Partial Least Squares Discriminant Analysis (sPLS-DA), both urine and serum metabolome profiles were significantly different between the two groups with a total of 29 discriminating metabolites in urine and serum. Fourteen of them were implicated in the nitrogen metabolism pathway including, gamma-aminobutyrate, 3-methyl-l-histidine and xanthosine (less abundant in SB compared with placebo group, all *p* < 0.05), adenine, aspartate and proline (more abundant in SB compared with placebo group, all *p* < 0.05). Metabolic pathways pointed to proline synthesis, a crucial component in collagen synthesis and osteoarticular system development. Urinary proline abundance was positively correlated with EGR (r = 0.45; *p* < 0.001). These findings highlight the potential benefits of maternal supplementation with SB promoting early neonatal growth, essential for the neonatal survival, through nitrogen metabolism orientation.

## 1 Introduction

The maternal-fetal relationship plays a pivotal role in shaping the development and health of offspring. According to the fetal programming theory initially proposed by Barker (1), factors and experiences during pregnancy may induce lasting modifications in the fetal anatomy, physiology, and metabolism, potentially contributing to the development of diseases in childhood and even in adulthood.

In the early stages of life, dogs encounter a spectrum of health challenges. One of the major concerns that puppies face during the 1^st^ months of life is growth problems. For instance, the early growth rate (EGR) is strongly associated with neonatal mortality (2). Indeed, the first 2 days of life are crucial for the newborn dog, as they reflect the adaptation to the extrauterine life but also the colostrum ingestion.

As all of the mentioned phenomena are strongly related to the dam, one could hypothesize that acting on the maternal organism could improve the newborn's health. Indeed, studies in humans and pigs demonstrated the possibility of modulating offspring growth through maternal nutrition during gestation and lactation (3, 4). One of the strategies of maternal programming via nutrition aiming to improve neonatal outcomes is maternal probiotic supplementation during gestation.

Probiotics are live microorganisms that, when administered in adequate amounts, confer a health benefit on the host (5–8). Among probiotics, *Saccharomyces cerevisiae* var. *boulardii* (SB) is a yeast extracted from lychee and mangosteen fruit that has been shown to have beneficial effects on digestive health, general immunity in humans (9) and on mental health in rats (10). In addition to effects observed on the host, its potential role as a tool for maternal nutritional programming on the offspring's health has been demonstrated recently. In pigs, supplementation of sows with SB during gestation and lactation modifies the intestinal microbiota of piglets, specifically increasing *Lactobacillus* species abundances, known to be beneficial for the host's health (11). The positive influence of SB supplementation in pregnant sows has been highlighted on the immunological status and response to stress of the newborn at birth, via a decreased blood cortisol concentration and increased white blood cell counts (12). To date, the effect of the maternal nutritional programming by yeast has never been investigated in the canine species.

The objectives of this study were thus (1) to evaluate the effect of live yeast SB supplementation of the pregnant and lactating bitches on EGR, urine and serum metabolome of their puppies at 2 days of life and (2) to study the relationship between these two elements.

## 2 Materials and methods

### 2.1 Ethics statement

This study was reviewed and approved by the local ethical committee (Ethics Committee for Animal Experimentation, Animal Science and Health No. 115; reference number: SSA_2020-004, Toulouse, France).

### 2.2 Animals, diet, and experimental design

#### 2.2.1 Dams

Of the thirty-six bitches included in the previous study by Guarigues et al. (13), 17 were selected for our study, to balance the cohort, ensure homogeneous groups, and healthy puppies (Table 1). Theses female dogs of medium-sized breed (Australian Shepherd, body weight between 15 kg and 25 kg) and large-sized breeds (Golden Retriever, Labrador Retriever, White Swiss Shepherd; body weight between 25 kg and 45 kg) were included within one breeding kennel and housed individually in the same conditions. No dam presented any health trouble at the time of inclusion.

**Table 1:** Characteristics of the 17 dams included in the study at the 28th days of gestation.

	**Placebo (*n =* 7)**	**SB (*n =* 10)**
	***n***	***n***
**Breed**		
Medium	3	4
Large	4	6
	**Mean** ± **SD**	**Mean** ± **SD**
Body weight (kg)	26.3 ± 4.5	25.4 ± 5.0
Age (years)	3.2 ± 1.2	3.3 ± 1.4
Parity	2 ± 2	3 ± 2
Body condition score^*^	4 ± 1	4 ± 1
Fecal score^#^	3 ± 1	3 ± 1

SB, Females supplemented with *Saccharomyces boulardii*.

* Body condition score evaluated on 9-point scale according to Laflamme (41).

# Fecal score was evaluated on 5-point scale (https://my.royalcanin.com/UserFiles/Docs/Digital-Toolkit/gi-fecal-scoring-chart-dog.pdf) on the basis of 3 consecutive days.

Ovulation was identified using a progesterone assay, with progesterone levels ranging between 4 ng/mL and 10 ng/mL. Then two natural matings were conducted on days 1 and 3 post-ovulation. Pregnancy diagnosis was carried out between days 25 and 30 post-ovulation using abdominal ultrasonography.

At the 28th day of gestation (G28), bitches were randomized according to their breed size, breed, age, parity, body condition score and fecal score and subsequently, they were allocated into two groups: placebo (*n* = 7) or supplemented group (*n* = 10; SB group) (Details in Table 1).

#### 2.2.2 Diet and probiotic supplementation in dams

Two extruded foods manufactured by CRUSTY FOOD SAS (Montardit, Verteuil d'Agenais, France) and formulated to meet NRC recommendations (14) were provided during the study: The first diet (Diet 1; Table 2) was given from the date of ovulation until the G28 and the 2^nd^ diet from G28 until the end of the study (56 days *post-partum*; Diet 2). Vitamin A, vitamin D3 and vitamin E were added as nutritional feed additives (Table 2) (15). All dogs had *ad libitum* access to water for drinking during the whole experiment.

**Table 2:** Nutrient composition of diets used in the study.

		**Feed**	
		**Diet 1**	**Diet 2**
Nutrient content (on raw mater basis)	Moisture, %	5.7	6.3
	Crude ash, %	8.7	9.3
	Crude protein, %	30.8	28.4
	Ether fat, %	15.1	16.8
	Crude fiber, %	2.7	<2
	Carbohydrates^a^, %	37.0	37.7
Feed additives	Vitamin E, mg/kg	124	114
	Vitamin A, IU/kg	7,670	7,330
	Vitamin D, IU/kg	1,080	1,000
Metabolizable energy^b^, kcal/100 g		385.8	408.6

a Carbohydrate was calculated from equation: %Dry Matter – (%Ether extract + %Crude Protein + %Crude Ash + %Crude fiber);

b Metabolizable energy was calculated from equation (14, 15). Diet 1: from ovulation until 27 day of gestation. Diet 2: from the 28 day of gestation until the end of the study.

The additive tested was *Saccharomyces cerevisiae* var. *boulardii* CNCM I-1079 (Levucell SB^®^, Lallemand SAS, Blagnac, France) given in 400-mg vegetal capsules (hydroxypropylmethylcellullose (HPMC) caps size 1 clear, Suheung Co, South Korea) containing 6.25% additive (YEAST, measured at 6.4 × 108CFU of additive/capsule), 92.75% potato starch and 1% E551a (silicic acid, precipitated and dried). Capsules were fed twice per day, in the morning and the evening, in a bullet of wet pet food to ensure full consumption. Each dog received 1.3 × 109 CFU of additive/day. The control group was fed the same capsules but without the additive (Placebo).

#### 2.2.3 Puppies

Among a total of 113 puppies born, 105 were born alive and only 81 puppies having all samples needed (urine and serum) were selected for this study. At birth, puppies were identified using a different colored collar, and their weight and sex were recorded. All included puppies were born naturally and remained healthy at the time of sampling (2 days *post-partum*). Puppies remained with their dams during the study period with unlimited access to maternal milk.

**Figure 1:**
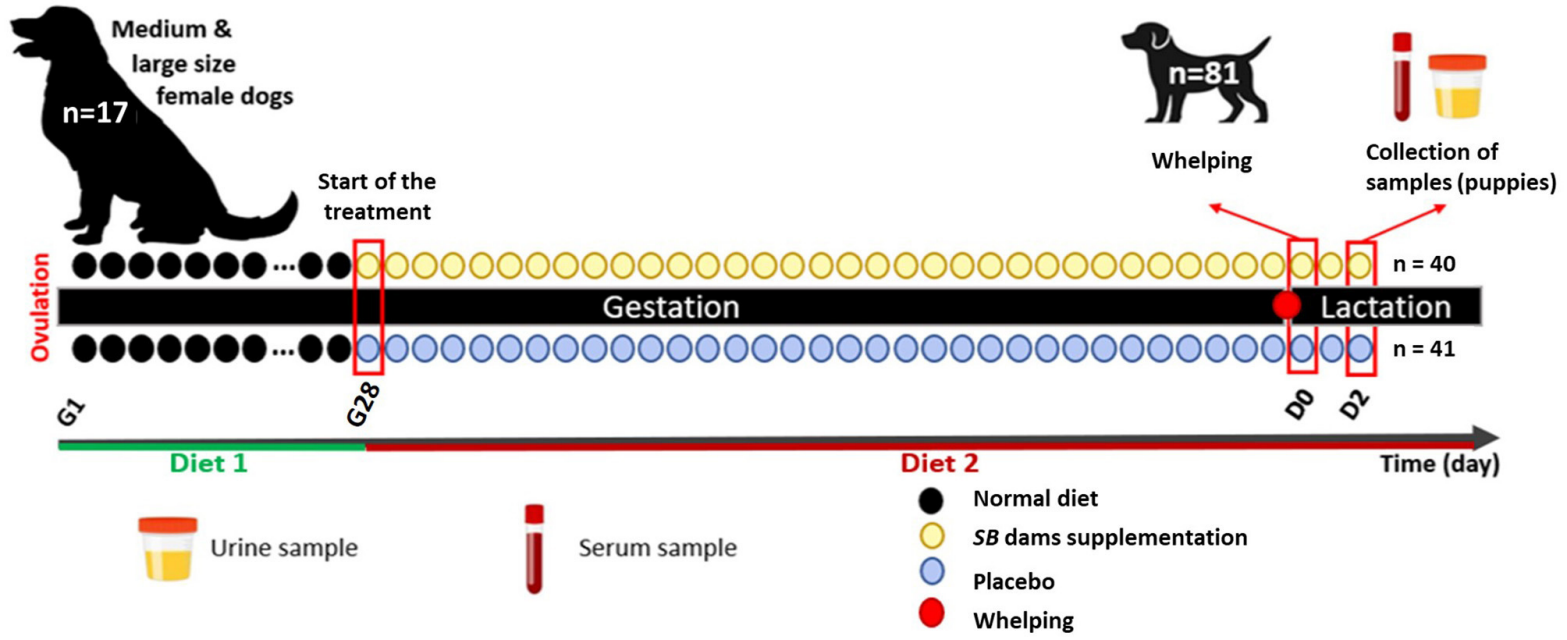
Experimental design of the study.

Included puppies underwent weight measurements at the minimum 0 and maximum 12 h following birth (D0). About 24 h later (D1) all puppies were examined and their rectal temperature and glycemia were measured to evaluate their health status. Puppies were weighed again 24 h later (D2), and at the same time urine and serum samples were taken as follows (Figure 1): about 1 mL of urine was obtained by gentle swabbing of perineal area (to stimulate spontaneous miction) and blood was collected from the jugular vein (1 mL), both on Eppendorf tubes. The blood was centrifugated (16,000 RCF for 10 min) to isolate the serum, and aliquot was stored at −20°C for 2 weeks and then transferred to −80°C until analysis.

### 2.3 Serum and urine metabolome analyses

Urine and serum metabolome analyses and data pre-processing were conducted at the BIOMET platform attached to the cardiovascular and nutrition research center (C2VN) located in Marseille, France.

### 2.4 Samples preparation

#### 2.4.1 Urine

The tubes were thawed on ice, then vortexed for 1 min. The samples were then centrifuged at 4°C for 15 min at 16,000 RCF. Following this, 50 μL of urine was drawn into 2 mL Eppendorf tubes and 400 μL of deionized water was added. After vortexing the mixture for 30 seconds, another round of centrifugation was performed. A total of 350 μl of the supernatant was collected in a 0.45 μm filter, then recentrifuged (4°C, 15 min 16,000 RCF). Then, 50 μL of the supernatant was collected in a glass vial with the insert then stored at −80°C before analysis by liquid chromatography–mass spectrometry (LC-MS).

#### 2.4.2 Serum

The tubes were thawed on ice then vortexed for 1 min. Then 50 μL of the sample was taken and 200%L of cold methanol (MeOH) (−20°C) was added. The mixture was slowly agitated for 1 min then incubated in the freezer (−20°C) for 1 h, vortex mixed for 30 seconds then centrifuged (4°C, 15 min, 11,000 RPM). A total of 200 μl of the supernatant was collected in a 10 kDa filter then recentrifuged (4°C, 45 min, 16,000 RCF). Following this, the tubes were evaporated under a stream of nitrogen then the dry extract was taken up in 120 μL Water/acetonitrile (90/10, v/v +0.1% formic acid) then vortexed for 1 min. The mixture was then transferred to a 0.45 μm filter tube and centrifuged for 15 min (4°C, 16,000 RCF). Then, 50 μL of the supernatant was collected in a glass vial with the insert then stored at −80°C before analysis by LC-MS.

For both sample types (urine and serum), two controls for each were carried out: the pool (quality control samples) as a mixture of 20 μL of each serum sample, urine sample and a blank samples, prepared the same way as the samples by taking 50 μL of deionized water.

### 2.5 LC-MS analysis

The LC-MS analysis and data treatment were performed as descried by Garrigues et al. (13). Briefly, the metabolic profile of the samples was acquired using a high-performance liquid chromatography (HPLC) system, a Thermo Fisher Scientific Ultimate 3,000 Series, coupled with a Q-Extractive Plus, a high-resolution mass spectrometer (Thermo Fisher Scientific). Chromatographic separation was achieved using hydrophilic and reverse phase chromatography in order to largely cover the metabolome identification. A mass spectrometry data were achieved in positive and negative ionization modes with a resolving power set to 35,000 full width at half maximum (FWHM). A tandem mass spectrometry spectra (MS/MS) were acquired using a Data Dependent Analysis experiment, at 17,500 FWHM resolving power.

Samples were analyzed randomly in an analytical batch and in the LC-MS system applying the quality control procedure. MS/MS spectra were acquired on a pooled sample for the metabolite annotation and identification.

### 2.6 Data pre-processing

Full scan spectra were converted to a mzXML format using Proteo Wizard and pre-processed using XCMS library script under R language. The following parameters were applied: peak picking: CentWave, peak grouping: density, retention time correction: obiwarp. Analytical batch was corrected using Van-der-Kloet algorithm and further filtering was applied on the extracted signals, such as removal of signals with coefficient of variation higher than 30% in quality control samples, and removal of noise signal.

Signal annotation was achieved using an “in-house” database with information about *m/z* and retention times in reverse and hydrophilic phase of nearly 800 metabolites. A match between the experimental and database information was done on the Galaxy Workflow 4 metabolomics platform using following conditions: m/z accuracy limit was set to 5 ppm, and retention time drift up to 0.7 min. After an automatic annotation, each annotated feature was visually verified and the annotation confirmed by fragmentation spectra comparison.

From this annotation, a table is obtained containing 205 identified/annotated metabolites with their intensities detected in the urine samples and 228 metabolites for serum. Subsequently, probabilistic quotient normalization (PQN) was carried out on each of the matrices.

### 2.7 Statistical analysis

All statistical analysis was performed using R software, version 4.2.1. (16).

#### 2.7.1 EGR

The early growth rate was calculated for each individual as follows: (weight D2 – weight D0) × 100/weight D0. An analysis of variance (ANOVA) was then performed to evaluate the effect of treatment, breed and litter size on growth rate, using the model:


$$\begin{array}{l} \text{EGR }=\text{ μ }+\text{ treatment }+\text{ breed }+\text{ litter size }+\text{ sex ε} \end{array}$$

#### 2.7.2 Metabolome

As metabolomes are compositional data (17), we firstly applied the Geometric Bayesian-multiplicative method (GBM) to handle the zero values, assuming that the probability of detecting the metabolite in the samples is not zero. The cmultRepl function from the zComposition package (18) was employed to perform this method. Then, using the abundance table generated from the GBM output, the composition package was utilized to transform the data into the Centered Log Ratio (CLR). Subsequently, after obtaining the CLR output, the effects of sex, mother, breed, and litter size were visually assessed using PLS-DA. No significant differences were observed for sex. A linear model was applied to identify the impact of the remaining fixed effect on each metabolite, using the following formula:


$$\begin{array}{l} \text{Formula }1\;:\text{ y}1\;=\text{ μ}1\;+\text{ mother }+\text{ breed }+\text{ litter size }+\text{ ε}1 \end{array}$$

*P-*values were obtained and used to calculate the percentage of metabolites affected by each confusion effect. The results revealed that the breed effect impacts 50% of the metabolites, while the maternal effect and litter size affect approximately 20%. Notably, the correction for the breed effect eliminates the influence of the maternal and litter size effects. Consequently, we opted to correct for the breed effect and proceeded to retrieve the residual data table for subsequent multivariate analyses:


$$\begin{array}{l} \text{Formula }2\;:\text{ y}2\;\sim \text{ μ}2\;+\;\left(1|\text{ breed}\right)\;+\text{ ε}2 \end{array}$$

The residuals derived from the final model were employed for the following analyses. Utilizing the corrected data matrix obtained from Formula 2, we employed the “tune.splsda” function to determine the optimal number of principal components (ncomp) and the number of variables (KeepX) per component for achieving the most effective discrimination between the two groups (Placebo and SB). Subsequently, a sparse Partial Least Squares Discriminant Analysis (PLS-DA) was performed using the “splsda” function from the MixOmics package (19), with the parameters (ncom and KeepX) recommended by “tune.splsda.” Evaluation of error rate was conducted through the utilization of balanced error rate (BER) analysis and area under the receiver operating characteristic curve (AUROC) was performed to assess the classification performance. Subsequently, PlotLoadings and variable importance in projection (vip) functions were employed on the principal components selected (as determined by “tune.splsda” function), in order to extract the relevant variables that discriminate our groups.

Metabolite set enrichment analysis was performed using Metaboanalyst 5.0 (20). website in order to identify significant urine and serum metabolic pathways. Correlations between the variables of different matrices were calculated based on multiblock sPLS-DA using the “network” function of the MixOmics package. A cut-off score of 0.50 was set in order to only represent variables whose scores are higher than the latter.

#### 2.7.3 Link between metabolome and EGR

Pearson's correlation with a threshold of 0.4 was used to highlight the links between individual metabolites and growth rate. Then, scatterPlot was produced using the ggplot2 package (21). The final diagram was produced using the BioRender website. Data are presented as mean ± standard deviation (SD).

## 3 Results

### 3.1 Characteristics of the included puppies

A total of 81 puppies alive at D2 (49 males and 32 females, 7 per litter on average) from 17 selected bitches were included in the study. Birth weights ranged from 214 g to 576 g with an average of 387g. At D1, the mean rectal temperature was 36.4 ± 0.8°C (min: 33.8°C – max: 38.3°C) and the glycemia was 120 ± 30.6 mg/dl (min: 64 mg/dl – max: 207 mg/dl) with no sign of diseases.

Among the included puppies, 49% (40 puppies) were born from 10 mothers supplemented with SB during gestation and lactation, while 51% (41 puppies) were born from 7 mothers supplemented with a placebo. Both groups of puppies were balanced according to the sex ratio, litter size, breeds, and birth weight (Table 3).

**Table 3:** Characteristics of puppies selected and included for metabolome analysis.

		**Placebo group**	**SB group**
Puppies (n)		41 (51%)	40 (49%)
Sex	Males (n)	27 (33%)	22 (27%)
	Females (n)	14 (18%)	18 (22%)
Litters (n)		7	10
Puppies fed by each lactating bitch (mean ± SD)		7 ± 2	8 ± 2
Puppies included per litter (mean ± SD)		6 ± 2	4 ± 2
Breeds (n)		4	4
Birth weight (mean ± SD)		395 ± 75 g	379 ± 68 g

Placebo group includes the following breeds: Golden Retriever (30% of puppies), Australian shepherd (46% of puppies), Labrador (10% of puppies), and Bernese Mountain dog (14% of puppies). The *Saccharomyces boulardii* group includes the following breeds: Golden Retriever (52% of puppies), Australian shepherd (35% of puppies), Labrador (5% of puppies), and Swiss White Shepherd (7% of puppies).

**Figure 2:**
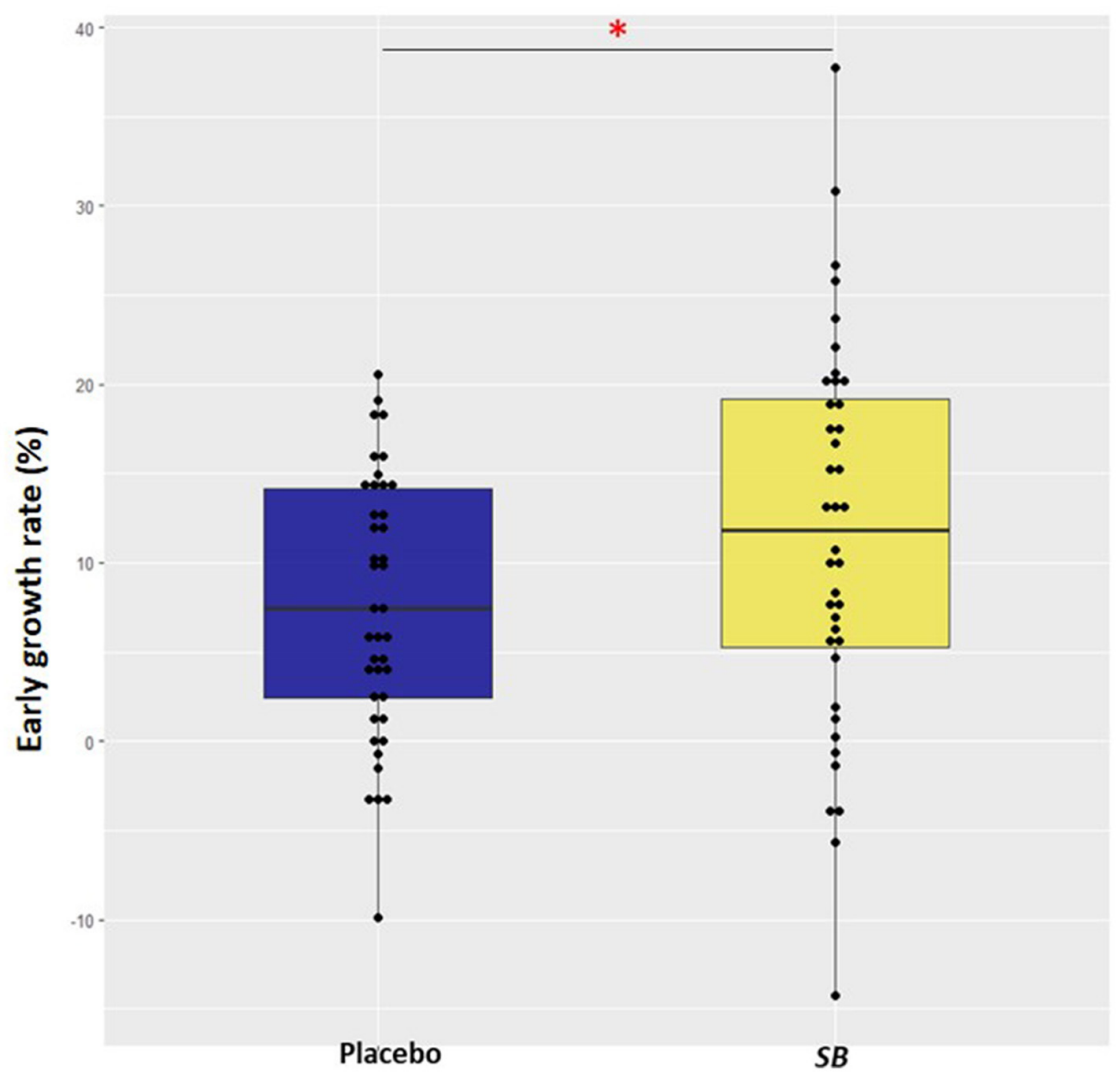
Box and whisker plot of early growth rate in puppies from mothers supplemented with *Saccharomyces boulardii* (*n* = 40; in yellow) during gestation and lactation and puppies from mothers supplemented with placebo (*n* = 41; in blue). Early growth rate (%) = (weight at 2 days - weight at birth)/weight at birth * 100. An ANOVA was carried out. One asterisk (*) indicates *p* value < 0.05.

Out of all puppies, EGR varied between −14.2% and 37.7%. According to the ANOVA test, the EGR was significantly higher in puppies from the SB group compared to those from the placebo group (+57%; *p*-value = 0.049) despite a high variability in the SB group (Figure 2).

### 3.2 Urinary and serum metabolome in newborn puppies

**Figure 3:**
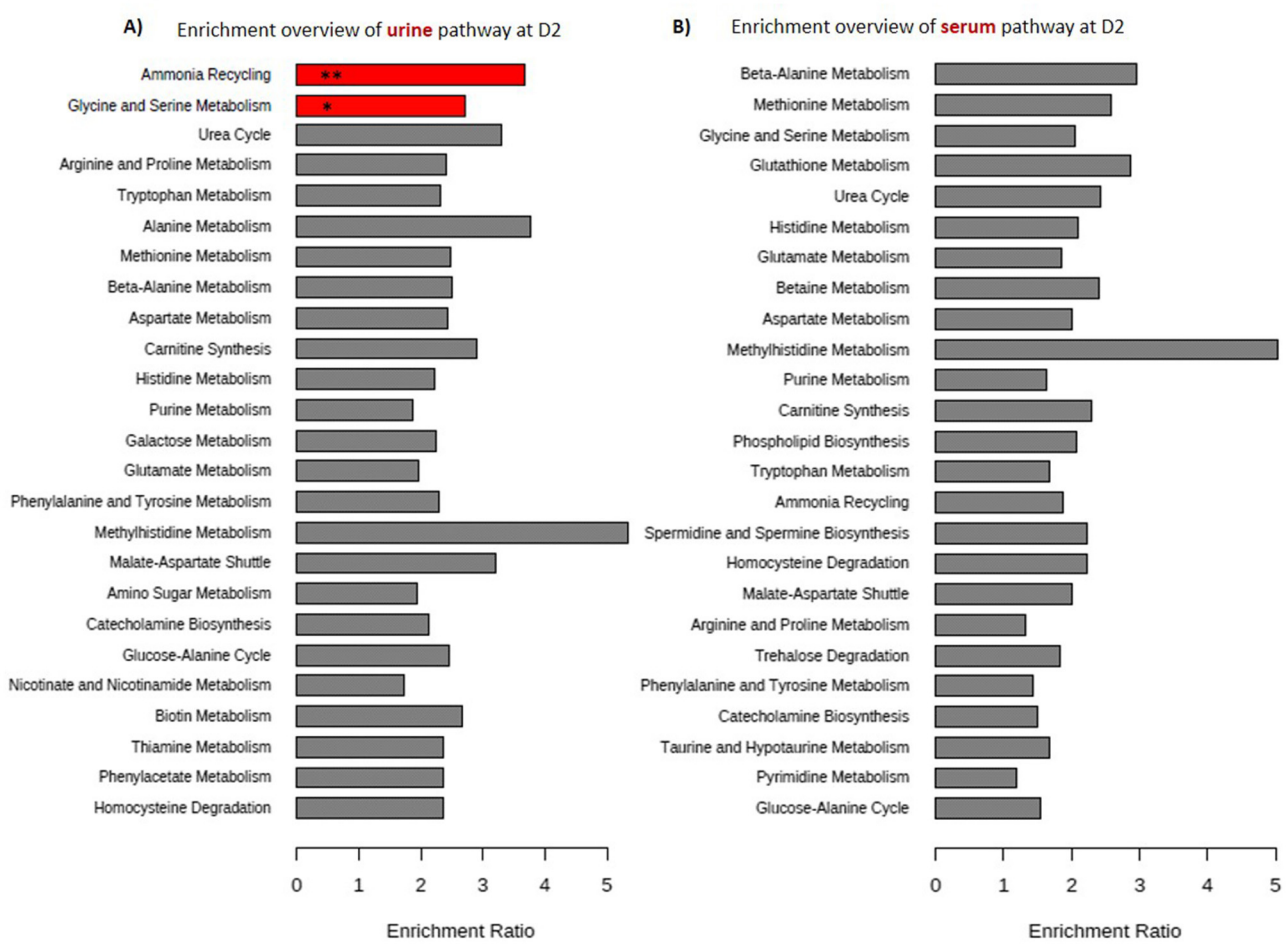
Summary of the enrichment analysis with the top 25 metabolic pathways obtained on 81 puppies at 2 days of age by Metaboanalyst 5.0. **(A)** Represents 206 urinary metabolites and **(B)** 228 serum metabolites. The enrichment rate was calculated by dividing the number of metabolites found by the number of metabolites expected for each pathway. The *P* value (P-adj) was calculated by hypergeometric test then a *post-hoc* Holm test was performed. In red are the metabolic pathways found significantly enriched after *p*-value correction. One asterisk (*) indicates *p* value smaller than 0.05, two asterisks (**) indicate *p* value < 0.01.

A total of 228 serum and 206 urine metabolites were detected in puppies at D2 according to the LC-MS analysis (Supplementary Table 1). The metabolites found in the urine and serum of neonates were for the vast majority part of the nitrogen metabolism pathway (Figure 3).

After the enrichment analysis and p-value correction, only the ammonia recycling and glycine-serine metabolism pathways were significantly overrepresented in urine samples (respectively, *p*-adj < 0.01, *p*-adj = 0.016; Figure 3), while no pathway was significantly overrepresented in serum samples.

### 3.3 Impact of maternal SB supplementation on the urinary metabolic profiles of newborn puppies

**Figure 4:**
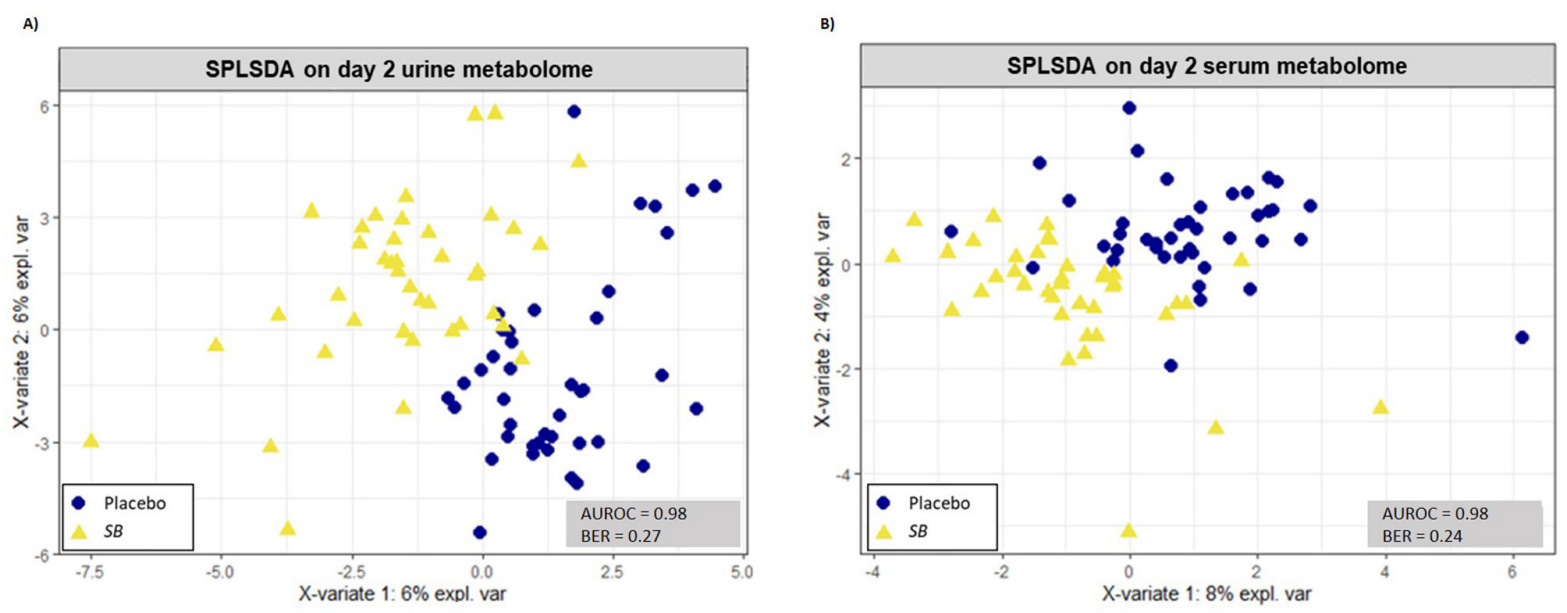
Sparse partial least-squares-discriminant analysis (sPLS-DA) of urine [model with 2 principal components and KeepX (30, 20)] **(A)** and serum [model with 2 principal components and KeepX (10, 5)] **(B)** metabolome in newborn puppies at 2 days of age coming from mothers supplemented with *Saccharomyces boulardii* (40 puppies; yellow triangles) or placebo (41 puppies; blue dots).

**Figure 5:**
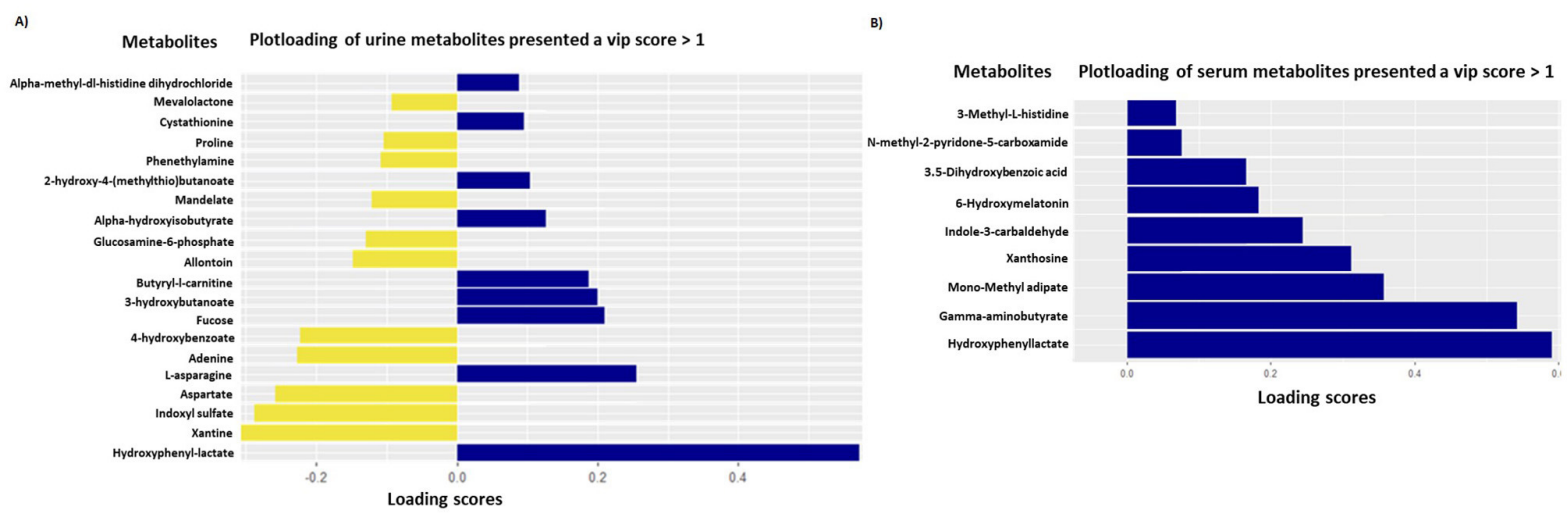
PlotLoadings on urine [model with 2 principal components and KeepX (30, 20)] **(A)** and serum [model with 2 principal components and KeepX (10, 5)] **(B)** metabolomes and their scores responsible for the discrimination between puppies from mothers supplemented with *Saccharomyces boulardii* (40 puppies; yellow bars) and those from the placebo group (41 puppies; blue bars).

The sPLSDA analyses performed on urinary metabolites allowed us to discriminate puppies of the SB group from those in the placebo group with a high accuracy of the model (AUROC ≥ 0.98 for urine metabolome). The two principal components explained 12% of the variance for urine metabolome and discriminated groups with an error rate (BER) of 0.27 (Figure 4A). On these principal components, VIP analyses allowed the selection of 20 out of the initial 206 urinary metabolites (Loading score comprised between −0.31 and 0.57), discriminating puppies of the SB from the placebo group (Figure 5A; Table 4). All these metabolites presented a VIP score >1.0.

**Table 4:** Uniquely distinctive urinary metabolites between *Saccharomyces boulardii* and placebo groups, based on Variable Importance in Projection (VIP).

**Urine metabolites**	**VIP**	**Values in SB compared to placebo**	**P-adj**
Hydroxyphenyllactate	7.12	↓	*p =* 0.000
Xantine	4.15	↑	*p =* 0.001
Indoxyl sulfate	3.92	↑	*p =* 0.001
Aspartate	3.59	↑	*p =* 0.002
L-Asparagine	3.54	↓	*p =* 0.002
Adenine	3.22	↑	*p =* 0.004
4-Hydroxybenzoate	3.19	↑	*p =* 0.004
Fucose	3.03	↓	*p =* 0.005
3-Hydroxybutanoate	2.93	↓	*p =* 0.005
Butyryl-l-carnitine	2.78	↓	*p =* 0.006
Allantoin	2.34	↓	*p =* 0.010
Glucosamine 6-Phosphate	2.12	↑	*p =* 0.013
Alpha-Hydroxyisobutyrate	2.09	↓	*p =* 0.014
Mandelate	2.03	↑	*p =* 0.014
Phenethylamine	1.89	↑	*p =* 0.017
Proline	1.85	↑	*p =* 0.017
2-Hydroxy-4-(Methylthio)butanoate	1.84	↓	*p =* 0.018
Cystathionine	1.75	↓	*p =* 0.019
Mevalolactone	1.71	↑	*p =* 0.020
Alpha-methyl -DL-histidine dihydrochloride	1.68	↓	*p =* 0.021

Arrow (↓) means a lower abundance of metabolites and the arrow (↑) a higher relative abundance in *Saccharomyces boulardii* compared to the placebo group. *P*-adj was obtained using Anova and *post-hoc* Bonferoni test.

Significant differences were noted between the SB and placebo groups in the abundance of all discriminating urine metabolites. Specifically, within the purine metabolism pathway, the SB group exhibited a significant increase in the abundance of urine adenine and xanthine, accompanied by a significant decrease in urine allantoin compared to the placebo group. Regarding amino acid metabolism, the SB group showed a significant increase in urinary abundance of phenethylamine, aspartate, and proline. In contrast, urinary abundances of cystathionine and butyryl-L-carnitine and L-asparagine were decreased compared to the placebo group.

### 3.4 Impact of maternal SB supplementation on the serum metabolic profiles of newborn puppies

The sPLSDA analyses performed on serum metabolites also achieved high accuracy in discriminating puppies of the SB group from those in the placebo group (AUROC ≥ 0.98 for serum metabolome). Similarly, the two principal components explained 12% of the variance of serum metabolome and distinguished the groups with an error rate (BER) of 0.24 (Figure 4B). From these components, VIP analyses identified 9 serum metabolites out of the initial 228 (Loading score between 0.07 and 0.59), which differentiated the SB group from the placebo group (Figure 5B; Table 5). Each of these metabolites had a VIP score >1.0.

**Table 5:** Uniquely distinctive serum metabolites between *Saccharomyces boulardii* and placebo groups, based on Variable Importance in Projection (VIP).

**Serum metabolites**	**VIP**	**Values in *SB* compared to placebo**	**P-adj**
Hydroxyphenyllactate	8.91	↓	*p =* 0.000
Gamma-aminobutyrate	8.19	↓	*p =* 0.000
Mono-Methyl adipate	5.38	↓	*p =* 0.001
Xanthosine	4.70	↓	*p =* 0.001
Indole-3-carbaldehyde	3.68	↓	*p =* 0.002
6-Hydroxymelatonin	2.75	↓	*p =* 0.004
3.5-Dihydroxybenzoic acid	2.49	↓	*p =* 0.004
N-methyl-2-pyridone-5-carboxamide	1.14	↓	*p =* 0.007
3-Methyl-L-histidine	1.02	↓	*p =* 0.008

Arrow (↓) means a lower relative abundance of metabolites in *Saccharomyces boulardii* compared to the placebo group. *p*-adj was obtained using Anova and *post-hoc* Bonferoni test.

Significant differences were observed between the SB and placebo groups in the abundance of these discriminating serum metabolites. Notably, within the purine metabolism pathway, the SB group showed a significant decrease in serum xanthosine levels compared to the placebo group. Additionally, the SB group had decreased levels of serum gamma-aminobutyrate and 3-methyl-L-histidine compared to the placebo group.

### 3.5 Correlations between urinary and serum metabolites and early growth of puppies

**Figure 6:**
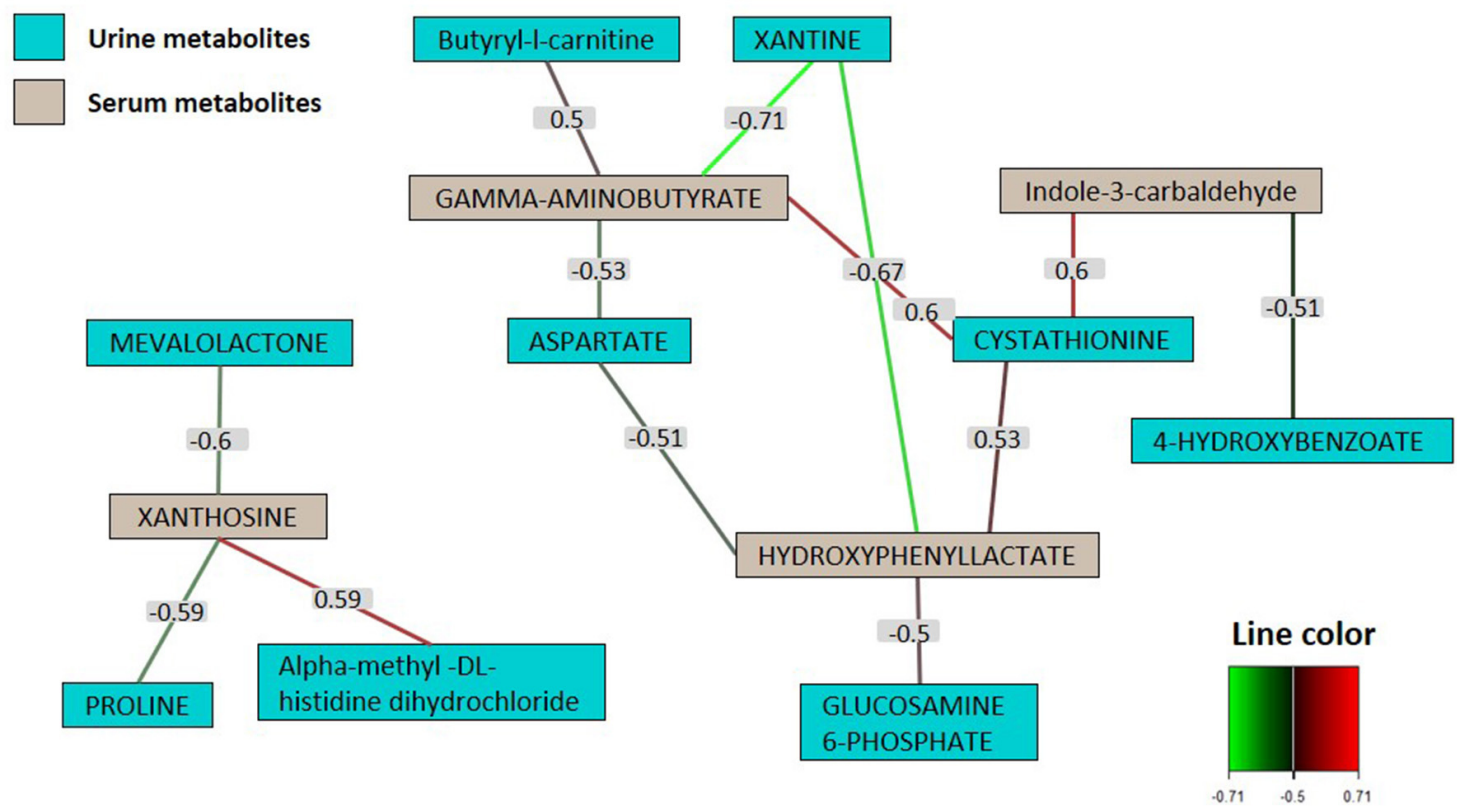
Correlation matrix of urine and serum metabolites selected by multiblock sPLS-DA. Network map of urine (in green) and serum (in gray) metabolites highly correlated (≥0.5). The number indicated on the line between nodules represents the value of the coefficient of correlation between two metabolites (only coefficients >0.5 were retained in the network analysis) (*n* = 81).

A network based on multiblock sPLS was used to investigate the correlation between urinary and serum metabolites (Figure 6). Correlation analysis was restricted to metabolites that exhibited significant differences between SB and placebo groups. The network exhibited a considerable degree of connectivity with each node being connected to several nodes. In line with the correlation score, two distinct correlation network groups were identified. Serum metabolites, particularly gamma-aminobutyrate and hydroxyphenyl lactate (both with a connectivity of 4), along with xanthosine (with a connectivity of 3), emerged as the most central, while urinary metabolites were peripheral. Xanthine presented the strongest (negative) correlations, in particular with hydroxyphenyl lactate (r = −0.67) and gamma-aminobutyrate (r = −0.71), followed by cystathionine positively correlated with indole-3-carbaldehyde (r = 0.60) gamma-aminobutyrate (r = 0.60) and hydroxyphenyl lactate (r = 0.53).

**Figure 7:**
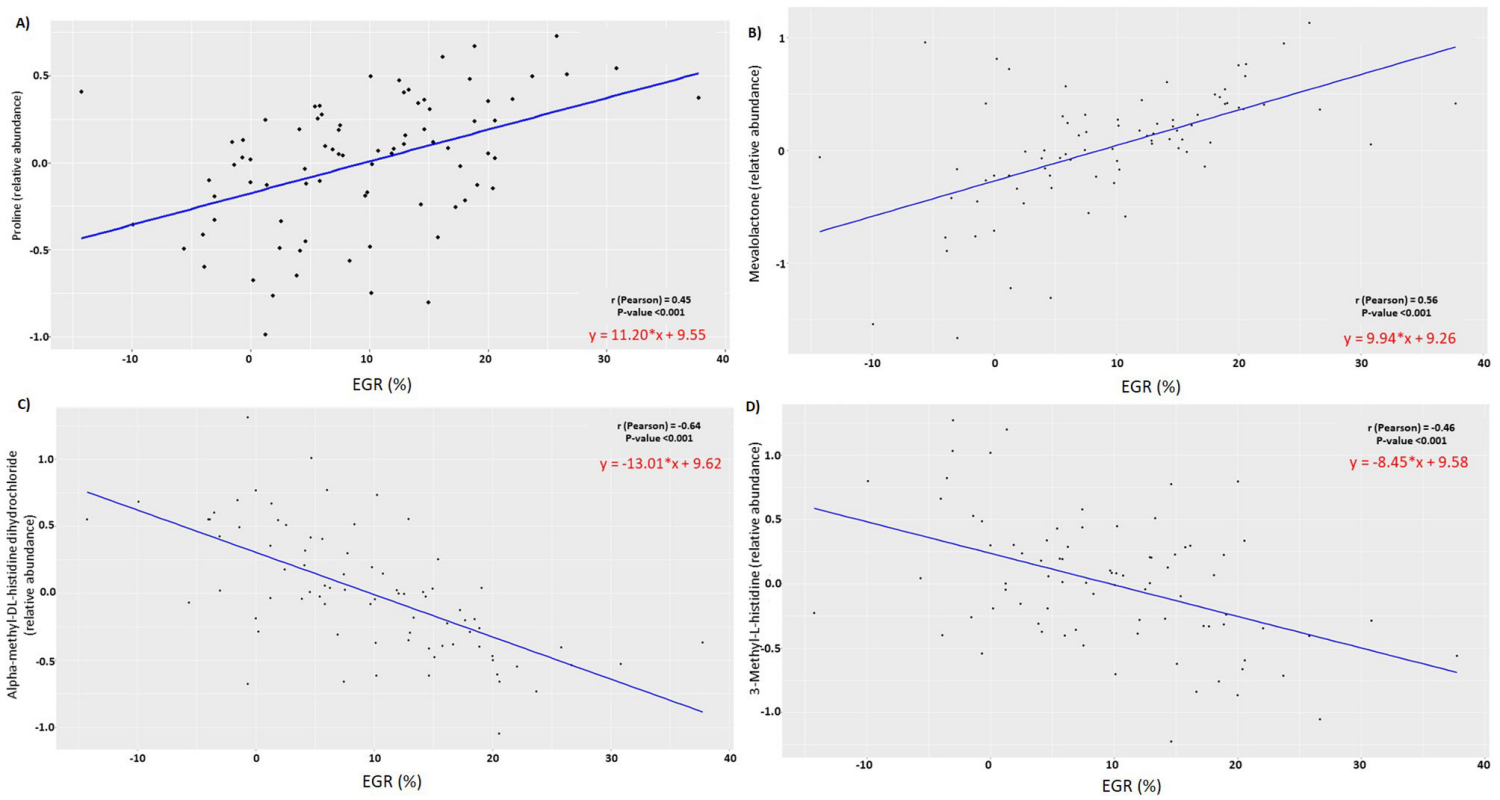
ScatterPlot of Pearson correlation between **(A)** Proline **(B)** Mevalolactone **(C)** alpha-methyl-dl-histidine dihydrochloride **(D)** 3-methyl-l-histidine relative abundances at 2 days of age and early growth rate (%) (*n* = 81).

According to Pearson's correlation analysis, the early growth rate was positively correlated with proline (r = 0.45) and mevalolactone (r = 0.56) in urine (Figures 7A, B), while it was negatively correlated with alpha-methyl-dl-histidine dihydrochloride (r = −0.64) in urine and 3-methyl-l-histidine (r = −0.46) in serum (Figures 7C, D).

## 4 Discussion

Although the interest in research on the neonatal period (i.e., the first 3 weeks of life) in the dog is continuously increasing, the canine neonatal morbidity and mortality rates remain relatively high (11%) (22). Several strategies are proposed to canine breeders and their veterinarians (23) to improve the newborn's health, mostly focused on whelping and suckling periods management. The modulation of offspring vitality and vulnerability through the mother during the gestation and lactation period is increasingly documented in humans and pigs (8), but very few studies focused on canine developmental programming through maternal nutrition in dogs. Our study demonstrated for the first time the interest of maternal supplementation with yeast during gestation and early lactation on the neonatal growth and metabolic profile, proposing a new strategy to breeders and veterinary practitioners.

The median growth rate during the first 2 days of life in the dog was estimated at about 3% in the study by Mila et al. (2). In our study, the growth rate in the placebo group was found to be twice as high at 7%. Despite this notable result, the SB group presented even a 1.5 higher growth rate over the first 2 days of life than puppies from the placebo group; however, there was a large variability among individuals (Figure 2).

In order to understand the mechanism explaining the increased growth in puppies born from SB mothers in our study, we decided to explore their urinary and serum metabolomes. Firstly, we investigated the different metabolic pathways found in the urine and serum of newborn puppies at 2 days of age. Our results showed that whether in urine or serum, the identified metabolites are mostly involved in nitrogen metabolism (amino acids and purines metabolic pathways, urea cycle, or even ammonia recycling cycle). Nitrogen metabolism is essential for cell division (24), which is important for the development of tissues and organs in puppies at this age. The need for nitrogen is the highest right after birth due to the intensive growth process and then decreases with age. In humans during the first 6 months, the daily protein requirement of infants is 2.2 g/kg, then this requirement gradually decreases to reach 0.75 g/kg/day in adulthood (25). The same pattern could be observed in puppies, supporting the overrepresentation of the nitrogen metabolism in puppies at 2 days of life in our study.

**Figure 8:**
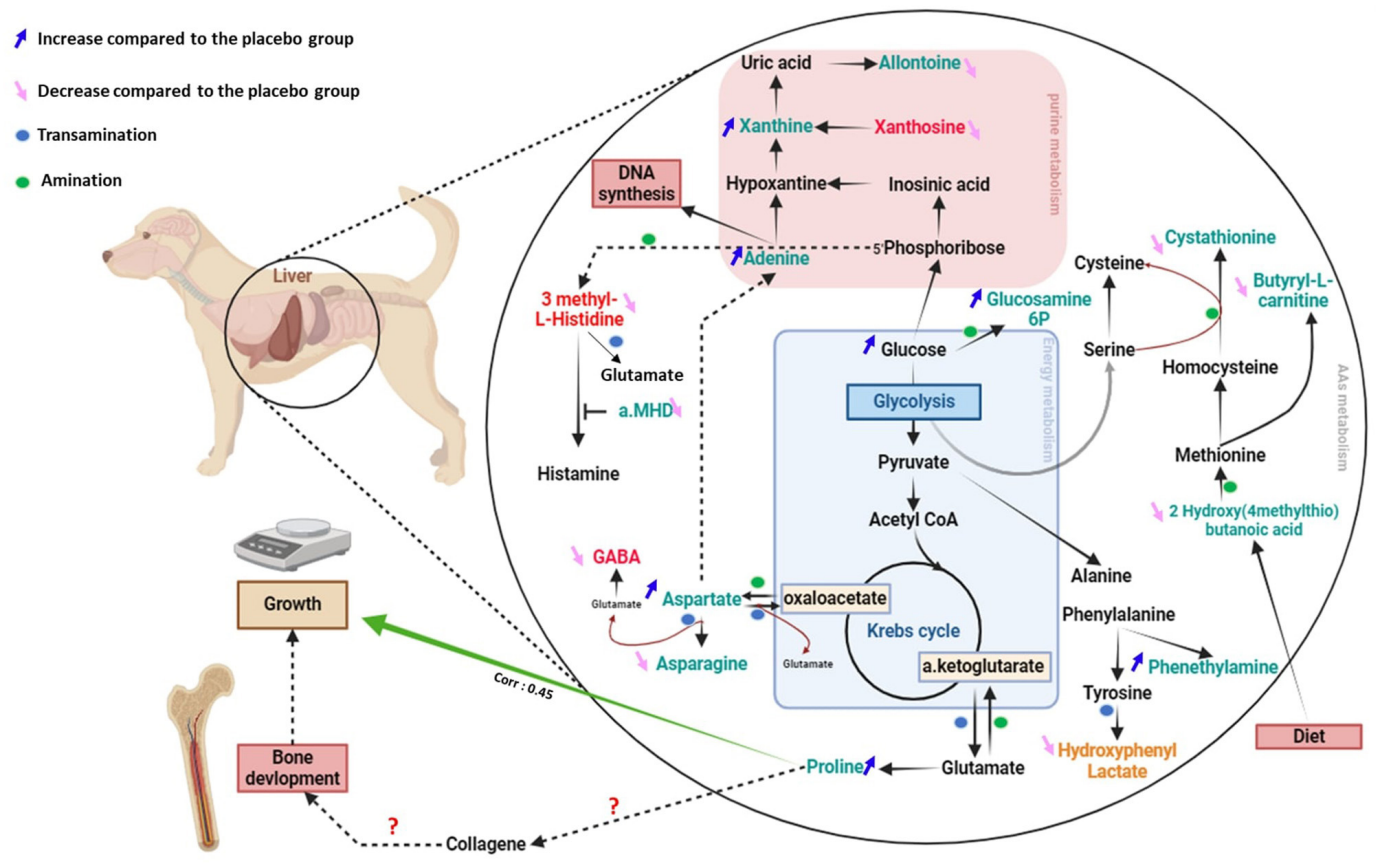
Diagram of the central pathways impacted in the serum and urine of puppies (at 2 days) by maternal supplementation with *Saccharomyces boulardii*. The arrow (↓) means a lower abundance of metabolites and the arrow (↑) a higher relative abundance in *Saccharomyces boulardii* compared to the placebo group. Urine metabolites are written in red, urine metabolites in green and metabolites found modified in both in orange (Hydroxyphenyl lactate).

**Figure 9:**
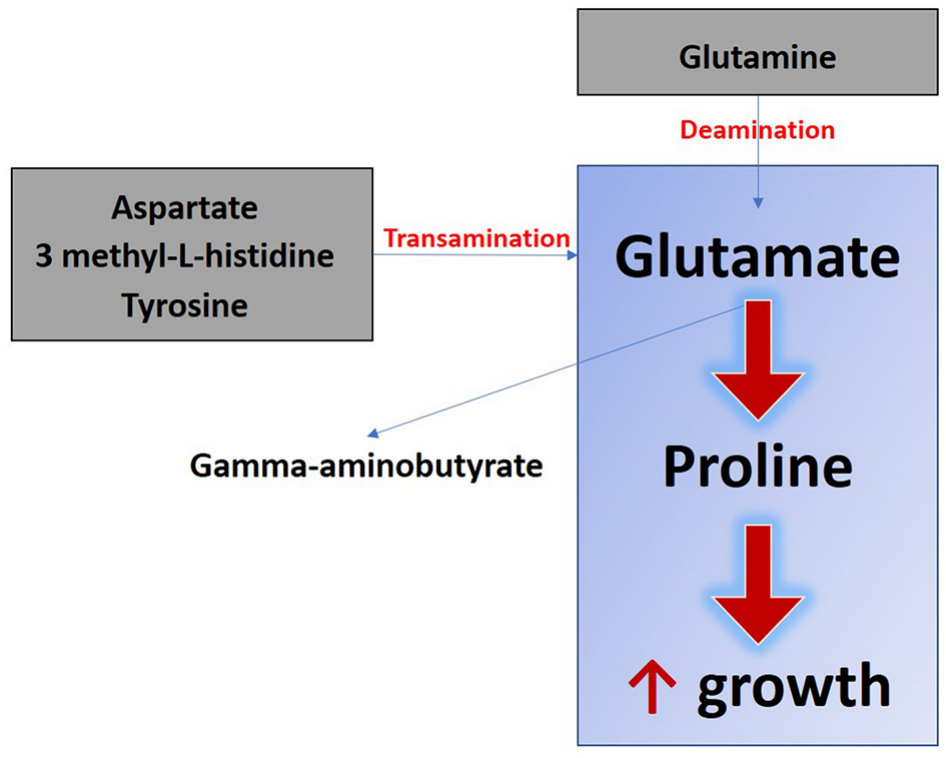
Schematic overview of the metabolic pathway influenced by maternal *Saccharomyces boulardii* supplementation during gestation and lactation that could explain enhanced growth in offspring. The metabolites involved in the synthesis of glutamate by transaminations and deaminations are represented in gray and the glutamate-proline-growth axis in the blue rectangle.

Secondly, we aimed to explore how maternal SB supplementation during gestation and lactation affect the metabolome of puppies. We have demonstrated that maternal yeast supplementation modifies the metabolic profile of offspring as observed in piglets (12). A total of 29 metabolites from serum or urine were impacted by the supplementation, 14 were amino acids, precursors, or products of their degradation, nucleotides, and energy metabolites. The metabolic pathways in which these metabolites are involved as well as their relationship were compiled in Figure 8. This figure shows that two major nitrogen metabolic pathways are modified thanks to the addition of SB: amino acids and purine metabolic pathways. The main changes are observed in reactions involving amination and transamination, in particular those using the glutamine/glutamate system. The glutamine/glutamate system is essential for protein anabolism and nucleotide synthesis, because of its ability to donate or take up an amine group (26, 27). The transformation of glutamine by deamination and the conversion of aspartate, histidine, and tyrosine by transamination are crucial processes for the biosynthesis of glutamate, an essential precursor of gamma-aminobutyrate and proline (28) (Figure 9). One of the most significant findings in our study is the increased urinary levels of proline and reduced levels of gamma-aminobutyrate observed in the SB group.

Proline is a crucial component in collagen synthesis and osteoarticular system development (29). This finding is particularly significant in light of the observed increased growth rate of puppies from mothers supplemented by SB. Interestingly, we also found a significant correlation between urinary proline abundance and growth rate in puppies. A similar positive correlation between blood proline concentration at 12 months and growth between 6 and 12 months was observed in babies, growth being evaluated using weight-for-height ratio and length velocity (30).

In parallel to this change in amino acid metabolism after SB maternal supplementation, we hypothesized a modification of the purine metabolism pathway in puppies at 2 days of age (Figure 8). In the same way as proline, we have noted an increase in urine adenine abundance. This could reflect an increase of purine metabolism (31) aligning with the needs of rapid growth.

During the 1^st^ days of life it is known that the tissues of the body grow at different rates, with bone tissue development being prioritized over other tissues, including the nervous system (32). Overall, according to here presented results, we hypothesize that yeast promotes glutamate utilization for proline synthesis, possibly at the expense of gamma-aminobutyrate production, which could be probably associated with accelerated osteoarticular growth in the supplemented newborns presenting increased overall growth rate values. Imaging studies must be conducted to confirm if these changes in nitrogen metabolism induced by maternal SB supplementation are linked to the bone development of puppies at this age.

This effect of maternal SB supplementation on changes in metabolites involved in the nitrogen metabolism pathway in the offspring can be elucidated through various mechanisms. First, maternal SB supplementation could modify the fetal environment during gestation with its effect observed postnatally. Indeed, maternal amino acids have the potential to modify fetal epigenetics, promoting growth and development (33). In a parallel study, we noticed that SB supplementation in late gestation led to a change in maternal metabolism, thus reinforcing the idea of fetal epigenetic modulation through the transplacental pathway (12). In the previous study mothers supplemented with SB had a higher blood level of metabolites involved in the metabolism of amino acids such as proline, tryptophan, beta-alanine, glycine, serine, and threonine during gestation compared to placebo group. We cannot conclude at this stage whether the changes in the nitrogen metabolism of the mothers have an influence on the nitrogen metabolism of the young, or whether these two changes are the result of the same cause.

In addition, changes in nitrogen metabolism could be influenced by maternal milk quality. In the study of Garrigues et al. (13), no significant protein modification was observed in the colostrum after SB supplementation of the bitches. However, breast milk contains also a fraction of non-protein nitrogen in the form of free amino acids, peptides, which could be ingested and used by newborns, playing an important role in their growth and development (34). A detailed analysis of the colostrum should be carried out to reach a conclusion.

Yeasts can influence the body in various ways, either indirectly by affecting the intestinal microbiota or directly by providing nutrients. Of all these possibilities, the one that could explain at least part of our observations is its richness in vitamins B, including B6 (35, 36). Vitamin B6 acts as an essential cofactor for more than 100 enzymes playing a crucial role in catalytic reactions, including decarboxylations and transaminations (37). Vitamin B6 can be transmitted transplacentally to the fetus. Colostrum is rich in vitamins, including vitamin B6 (38). Intake of vitamin B6 transplacentally or via colostrum could play an important role in modulating nitrogen metabolism in newborns from SB supplemented dams (39). In future studies, it would be interesting to evaluate the effect of maternal SB supplementation on the abundance of vitamin B6 in the plasma and colostrum of female dogs and its link with offspring metabolome and growth changes.

Moreover, the study by Garrigues et al. (13) demonstrated in dogs that maternal supplementation with SB altered the composition of the maternal gut microbiota, increasing the abundance of fiber-degrading bacteria while reducing undesirable bacteria like *Campylobacter*. Maternal intestinal microbiota during gestation and lactation plays a crucial role in shaping the gut microbiota of newborns (40). Modulating the maternal microbiota through SB during gestation could positively influence the microbial colonization of the newborn, potentially impacting their metabolome and growth. This hypothesis remains to be demonstrated in further studies.

These findings highlight the potential benefits of maternal supplementation with SB in promoting offspring growth potentially through metabolic nitrogen metabolism regulation. However, this pathway requires further investigation, in particular taking into account the effect of the age and time of sampling non-studied in this work. Additionally, more research is needed to investigate whether there is a dose-dependent effect of SB and explore the long-term implications of these modifications on the overall health and development of the offspring.

## Data Availability

The datasets presented in this study can be found in online repositories. The names of the repository/repositories and accession number(s) can be found in the article/Supplementary material.

## Supplementary material

Supplementary Table 1 Raw data of the urinary and serum metabolome of puppies, categorized by maternal supplementation group.
